# The dual roles of autophagy and the GPCRs-mediating autophagy signaling pathway after cerebral ischemic stroke

**DOI:** 10.1186/s13041-022-00899-7

**Published:** 2022-02-02

**Authors:** Weichen Hou, Yulei Hao, Li Sun, Yang Zhao, Xiangyu Zheng, Lei Song

**Affiliations:** 1 Department of Neurology and Neuroscience Center, The First Hospital of Jilin University, Xinmin Street 71#, Changchun, 130021 China; 2 Department of Respiratory Medicine, Center for Pathogen Biology and Infectious Diseases, Key Laboratory of Organ Regeneration and Transplantation of the Ministry of Education, The First Hospital of Jilin University, Xinmin Street 71#, Changchun, 130021 China

**Keywords:** Ischemic stroke, Autophagy, Macroautophagy, G-protein-coupled receptors, Neuropharmacology

## Abstract

Ischemic stroke, caused by a lack of blood supply in brain tissues, is the third leading cause of human death and disability worldwide, and usually results in sensory and motor dysfunction, cognitive impairment, and in severe cases, even death. Autophagy is a highly conserved lysosome-dependent process in which eukaryotic cells removal misfolded proteins and damaged organelles in cytoplasm, which is critical for energy metabolism, organelle renewal, and maintenance of intracellular homeostasis. Increasing evidence suggests that autophagy plays important roles in pathophysiological mechanisms under ischemic conditions. However, there are still controversies about whether autophagy plays a neuroprotective or damaging role after ischemia. G-protein-coupled receptors (GPCRs), one of the largest protein receptor superfamilies in mammals, play crucial roles in various physiological and pathological processes. Statistics show that GPCRs are the targets of about one-fifth of drugs known in the world, predicting potential values as targets for drug research. Studies have demonstrated that nutritional deprivation can directly or indirectly activate GPCRs, mediating a series of downstream biological processes, including autophagy. It can be concluded that there are interactions between autophagy and GPCRs signaling pathway, which provides research evidence for regulating GPCRs-mediated autophagy. This review aims to systematically discuss the underlying mechanism and dual roles of autophagy in cerebral ischemia, and describe the GPCRs-mediated autophagy, hoping to probe promising therapeutic targets for ischemic stroke through in-depth exploration of the GPCRs-mediated autophagy signaling pathway.

## Introduction

Ischemic stroke is a devastating condition caused by a lack of blood supply to the brain, usually resulting in sensory and motor dysfunction, cognitive impairment, and in severe cases, even death [1, 2]. About 795,000 of new or recurrent strokes occur every year, which is the third leading cause of human death and disability worldwide that endangers the health of middle-aged and elderly people [1]. Identification of novel therapeutic targets is a critical unmet need of the field. Autophagy is a highly conserved lysosome-dependent degradation system in eukaryotes that promptly responds to energy supply and insufficient nutrients [3–5]. Autophagy can facilitate the clearance of aggregated proteins and damaged organelles under stress conditions [3, 4, 6, 7]. A consensus has yet to be reached on the effects of autophagy in ischemic stroke, which has arisen widespread interest in clarifying the dual roles of autophagy after ischemic stroke.

G-protein-coupled receptors (GPCRs), one of the largest protein receptor superfamilies in mammals, play critical roles in many physiological and pathological processes [8, 9]. Nutritional deprivation can activate GPCRs directly or indirectly and mediate a series of downstream biological processes, including autophagy [8–10]. Several recent studies show that GPCRs play important roles in the induction and regulation of autophagy after ischemic stroke [11–13]. Since GPCRs are currently the most widely used drug targets, GRCRs-mediated autophagy signaling pathway in ischemic stroke may have potential neuropharmacological value. Therefore, this review will systematically discuss the dual roles of autophagy in cerebral ischemia and elaborate the GPCRs-mediated autophagy signaling pathway, hoping to find new treatment strategies for cerebral ischemia.

## Autophagy

The main physiological functions of autophagy include catabolism, removal of damaged or senescent cellular components, maintenance of genome stability, and immune regulation [4, 14], which improve cell adaptation ability to a certain extent through removing misfolding proteins, damaged organelles, and invasive microorganisms [3, 4, 15].

### Classification

Generally, autophagy can be divided into three types according to the way by which cytoplasmic substrates are transported to lysosome for degradation, including macro-autophagy, microautophagy, and chaperone-mediated autophagy (CMA) [4, 5, 16]. Microautophagy directly engulfs the target cargo into lysosome for lysosomal degradation through invagination or protuberance of the lysosomal membrane [17, 18]. CMA is extremely selective in facilitating lysosomal degradation of the KFERQ motif-containing proteins which can bind to the heat shock 70 kDa protein 8 (HSPA8/HSC70) chaperone [3, 19]. Macroautophagy mainly sequestrates the targeted cytoplasmic components in double-membrane vacuoles named autophagosomes, and then fuse with lysosomes to form autolysosomes to achieve the orderly lysosomal degradation [19, 20]. A large number of existing studies in mammalians have shown that macroautophagy plays an important regulatory role in a variety of physiological and pathological conditions, such as immunity [21, 22], cancer [23], aging [24], atherosclerosis [25, 26], neurodegenerative diseases [27], cerebrovascular diseases [28, 29]. Since macroautophagy is the most widely studied and the most concerned form of autophagy, we will then primarily focus on macroautophagy which is henceforth referred as “autophagy”.

### Selective autophagy

Initially, researchers reported that autophagy randomly sequestered the cytoplasmic components into autophagosomes, indicating that autophagy might be a non-selective process to a large extent [30]. Research on selective autophagy mechanisms has expanded rapidly over the last decade, and growing evidence suggests that many forms of selective autophagy exist [31, 32]. Under physiological or pathological conditions, damaged or excessively accumulated organelles, including mitochondria, peroxisomes [33, 34], lipid droplets [35], ribosomes and endoplasmic reticulum (ER) [36], can be specifically separated into autophagosomes through mechanisms mediated by a collection of specific autophagy-related molecules, which are separately named mitophagy, pexophagy, xenophagy, ribophagy and reticulophagy [31, 37].

Mitochondria are the most important organelles for energy metabolism and redox reactions [38]. Mitophagy can induce the removal of damaged or senescent mitochondria to maintain the function of mitochondria, which is extremely important for energy metabolism and cell homeostasis [39]. Recent studies have shown that mitophagy is involved in pathological mechanisms of many diseases, mainly including cerebral ischemic diseases, neurodegenerative diseases, cardiovascular diseases, malignant tumor, inflammation and autoimmune diseases [40–42].

### Autophagic mechanisms

Since autophagy-related genes and proteins were identified and named as *ATG* and Atg respectively in yeast [43], almost 30 *ATG* genes have been gradually confirmed [44]. Studies in higher eukaryotes have identified many Atg homologs [44]. The core machinery of autophagy mainly includes formation of the pre-autophagosomal structures (PAS) or phagophores, elongation and closure of the PAS membrane to form autophagosomes, autolysosomes formation and finally degradation of proteins and organelles [45].

Autophagy initiation is regulated by the ULK1-Atg13-FIP200 complex [46]. When nutrients are sufficient, the ratio of adenosine 5ʹ-monophosphate (AMP) to ATP decreases, which can induce mammalian target of rapamycin (mTOR) to inhibit unc-51-like kinase 1 (ULK1) and Atg13 [46]. Adenosine monophosphate-dependent protein kinase (AMPK) is the main positive regulator of autophagy. Nutrient deficiency or hypoxia increases the ratio of AMP to ATP, which induces AMPK to activate ULK1 [47].

Formation of PAS membrane is mediated by the PtdIns3K complex (PIK3C3/VPS34-beclin-1-Atg14) [48]. It enriches phosphatidylinositol 3-phosphate (IP3) at the initiation site to recruit regulatory proteins, such as Atg9, which expands the isolation membrane by collecting lipids. This stage is regulated by Akt (protein kinase B), AMPK, or ULK1. Phosphorylation of beclin-1 by Akt inhibits autophagy, while AMPK or ULK1 promotes the formation of the PtdIns3K complex [49, 50].

Elongation of the PAS membrane is regulated by Atg12–Atg5–Atg16L1 and LC3/Atg8-PE (microtubule-associated protein light chain 3-phosphatidylethanolamine) ubiquitin-like binding systems [51]. The 800-kDa Atg12–Atg5–Atg16L1 tetramers are located on the PAS membrane and participate in membrane elongation [45]. When the PAS membrane is closed, the Atg12–Atg5–Atg16L1 complex detaches and returns to cytoplasm [52]. LC3/Atg8-PE ubiquitin-like binding complex is another conjugated complex that attaches to the PAS membrane [53, 54]. The PAS membranes continuously extend and finally closed to form autophagosomes, meanwhile capturing misfolded proteins or damaged organelles into autophagosomes randomly or selectively. As lysosomes are usually located in the perinuclear region, autophagosomes are transported to lysosomes along microtubules.

Autolysosomes formation by fusion of autophagosomes and lysosomes constitutes the last stage. Subsequently, lysosomal enzymes effectively degrade the wrapped contents and release small molecules such as amino acids back to cytoplasm for catabolism.

### Autophagic flux

The dynamic process in which cargos are randomly or selectively captured by autophagosomes, transported and finally degraded in autolysosomes, is called autophagic flux. LC3-II and p62/SQSTM1, two important autophagy markers, are commonly used to analyze the integrity of autophagic flux. Increased LC3-II expression is usually due to an increase in autophagosomes or hindered degradation [55]. If the expression of LC3-II increases after lysosomal inhibitors treatment, it usually indicates that autophagic flux is intact. p62/SQSTM1 can promote the ubiquitination and subsequent degradation of targeted cargo molecules, increased p62/SQSTM1 expression may predict destruction of autophagic flux [56]. In addition, increased lysosomal enzyme activity (cathepsin D, acid phosphatase, and b-Nacetyl hexosaminidase) may also represent enhanced autophagic flux [57]. An intact autophagic flux is very important for cellular homeostasis, especially terminally differentiated and non-dividing cells [58]. Impaired autophagic flux can lead to excessive accumulation of autophagosomes, leakage of lysosomes, and even cell death [59].

## Autophagy and ischemic stroke

Ischemic stroke is mainly caused by cerebral blood flow blockage caused by cerebrovascular thrombosis, embolism or other reasons, leading to abnormal energy metabolism, sodium and chlorine influx, potassium efflux, cell membrane depolarization and cell edema. Subsequently, a series of damage cascades (calcium overload, excitatory amino acid toxicity, free radical generation, oxidative stress, inflammation and apoptosis) could trigger irreversible brain damage and result in a positive feedback loop that ultimately cause severe damage to neurons, glial cells, endothelial cells and their interconnections. In recent years, more and more studies have confirmed the important role of autophagy in the pathophysiological mechanisms after ischemic stroke.

### Underlying mechanisms of autophagy initiation in ischemic stroke

**Fig. 1:**
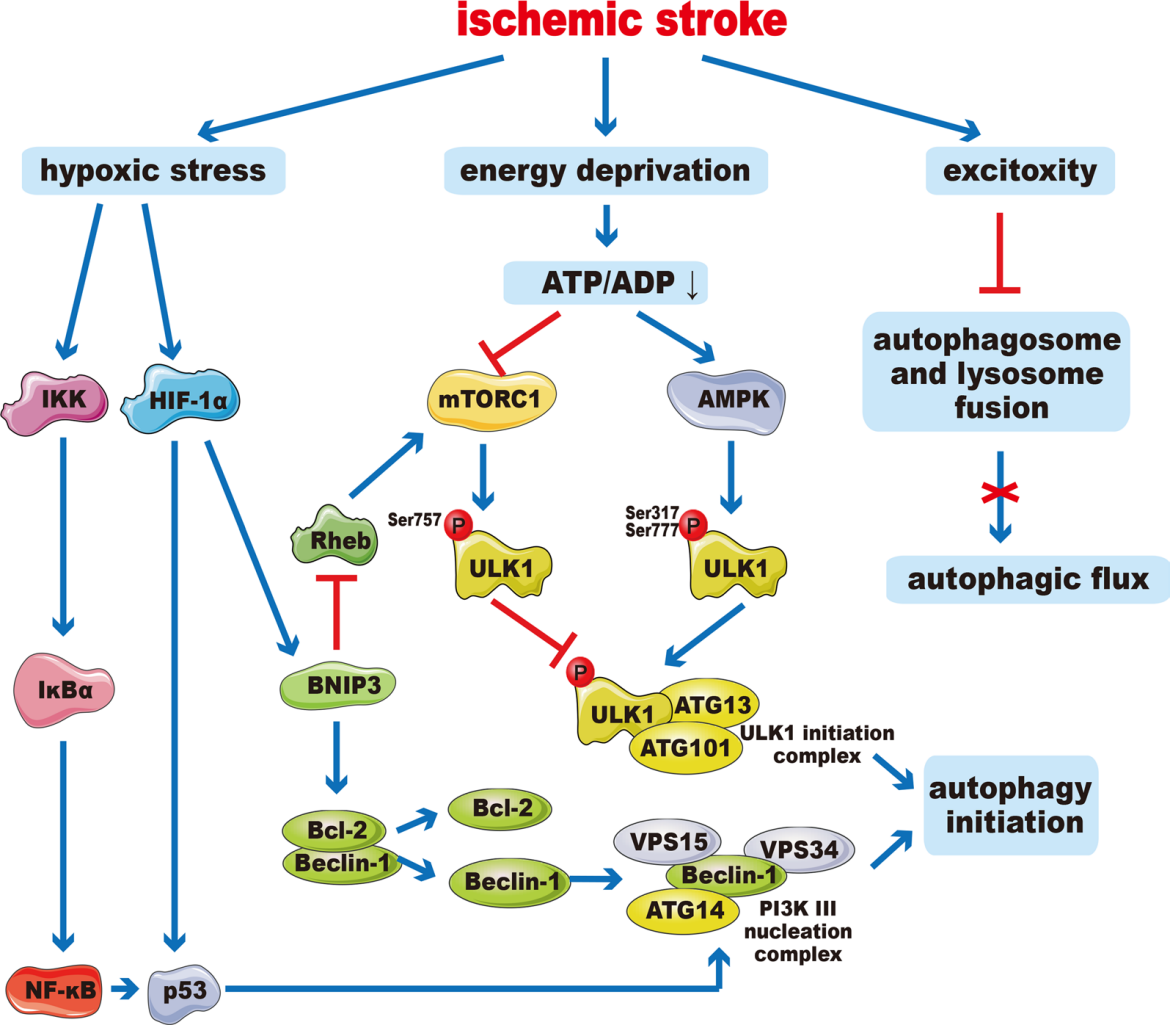
Core mechanisms of autophagy initiation in ischemic stroke. Energy failure could activate AMPK and inhibit mTORC1, which subsequently phosphorylates ULK1 and leads to ULK1 activation. Hypoxia promotes the production and release of HIF-1, which promotes the expression of BNIP3 that can mediate the dissociation of beclin-1 and Bcl-2. ULK1 activation and beclin-1 release can promote the formation of the PtdIns3K complexes and the ULK1-Atg13-FIP200 complexes that are both essential for autophagy initiation. NF-κB can also mediate autophagy initiation through p53 signaling pathway. Excessive excitotoxity prevents the fusion of autophagosomes and lysosomes, thereby inhibiting autophagic flux

Mounting studies gradually unveiled the mechanism of autophagy initiation after ischemic stroke (Fig. 1).

#### HIF-1a

Hypoxia inducible factor 1a (HIF-1a) is one of the key transcription factors activated by hypoxic stress, which can induce transcription of a variety of hypoxia-responsive genes, such as vascular endothelial growth factor, erythropoietin, glucose transporter 1, glycolytic enzymes [60, 61]. Bcl-2/adenovirus e1b-interacting protein 3 (BNIP3), an important target gene of HIF-1a, can activate autophagy by competing with beclin-1 to bind with Bcl-2, which mediates the dissociation of Bcl-2 and beclin-1 [62, 63]. BNIP3 can also inhibit Rheb, which then inhibits mTOR activity and activates autophagy [64]. HIF-1a can activate autophagy by up-regulating p53 after ischemic stroke [61]. It has been proved that hyperbaric oxygen could improve cerebral ischemic stroke injury possibly via inhibiting HIF-1 and subsequently inhibiting autophagy in rats MCAO model [65].

#### AMPK, mTOR

mTOR and AMPK are key molecules that responds to stress, which coordinate with each other and play crucial roles in autophagy initiation [62]. In case of sufficient energy supply, AMPK activity is inhibited, while mTORC1 phosphorylates ULK1 at Ser757, which inhibits ULK1 activation. Under insufficient energy supply conditions after ischemia, mTORC1 activity is inhibited, while AMPK phosphorylates ULK1 at Ser317 and Ser777, which triggers autophagy initiation [66]. Pretreatment with metformin (AMPK activator) can enhance autophagy by activating AMPK activity, resulting in reduced infarct volume, decreased nerve cell apoptosis, and improved neurological [67] deficits. Studies have shown that cerebral ischemic preconditioning can exert a neuroprotective effect by regulating AMPK to activate autophagy [68]. Since AMPK is the main energy regulator, studies on AMPK-dependent autophagy may reveal new treatment strategies based on improving energy metabolism [69, 70]. Activation of the α7 nicotinic acetylcholine receptor, a subtype of nicotinic acetylcholine receptors, could enhance autophagy partly through the AMPK–mTOR–p70S6K signaling pathway in neurons, rescuing neurons from OGD/R injury [71].

#### NF-kB

Nuclear factor-kappa B (NF-κB) encodes a variety of pro-inflammatory and pro-apoptotic proteins, which could exert key regulatory functions in inflammation and apoptosis [72]. Under ischemic conditions, IκappaB kinase (IKK) is activated and phosphorylates IκBa, then NF-κB is released and translocated into nucleus to mediate autophagy [73]. Knockout of NF-κB p50 subunit in mice can promote autophagy via inhibiting mTOR pathway after cerebral ischemia [74]. In addition, research results indicate that cathepsin B-dependent autophagy plays a key role in NF-κB activation [75].

#### Autophagic flux disruption

Autophagic flux disruption is an important reason for the abnormal accumulation of Atg proteins, such as LC3 and beclin-1 [76]. In neonatal rats with hypoxic-ischemic brain injury, the enhancement of autophagic flux can mediate neuronal apoptosis and autophagic cell death [77]. Studies have confirmed that ischemia-induced excitatory amino acid toxicity can disrupt autophagic flux in hippocampal neuronal cells, and enhancing autophagy can protect cells from excitotoxic death [78]. However, the lack of comprehensive studies on autophagic flux under various conditions limits our perception. Interestingly, recent research suggests a new role of autophagy, which is to reintegrate autophagosomes and mediate the extracellular secretion of unconditionally secreted proteins [37].

### Dual roles of autophagy in ischemic stroke

At present, many studies have confirmed autophagy activation after ischemic injury, promoting the clearance and reuse of cytoplasmic constituents. Regrettably, the results are controversial on whether autophagy exerts neuroprotective effects or exacerbates cerebral ischemia injury.

#### Neurons

Neurons, non-dividing and terminally differentiated cells, are extremely sensitive to ischemia and hypoxia and elimination of misfolding proteins and damaged organelles mediated by autophagy is extremely important to maintain cellular homeostasis. Autophagosomes formed in terminal regions of dendrites and axons must be transported and fuse with lysosomes distributed in perinuclear cytoplasm. Therefore, damage to dendrites and axons may cause failure in autophagosomes transportation and interrupted autophagic flux [79]. Under physiological circumstances, neuronal autophagy is maintained at a relatively low basal level, which are anti-apoptotic [58]. However, the exact role of neuronal autophagy after ischemic stroke is complicated and contradictory, and a consensus has not been fully reached.

Numerous studies have shown that neuronal autophagy after ischemic stroke is one of the important mechanisms of cerebral ischemia injury and closely related to neuronal death. For instance, the administration of autophagy inhibitor 3-methyladenine (3-MA) reduced apoptosis of PC12 cells exposed to serum deprivation, indicating that autophagy can cause neuronal damage or even death in vitro [80]. 3-MA administration also inhibits neuronal death in SH-SY5Y cells and primary cultivated neurons exposed to oxygen glucose deprivation (OGD). Intracerebroventricular injection of 3-MA 4 h after ischemia can significantly inhibit neuronal autophagy and reduce infarct volume in rats exposed to transient focal cerebral ischemia [70]. Down-regulation of beclin-1 or Atg7 with small interfering RNA (siRNA) can inhibit neuronal autophagy and reduce excitotoxicity in rats [55]. In rats MCAO model, intracerebroventricular injection of 3-MA and bafilomycin A1 (BFA-1) can significantly reduce cerebral infarction volume and cerebral edema, and improve motor function [81]. The neuroprotective effects of 3-MA and BFA-1 may be through inhibiting the up-regulation of LC3-II and cathepsin B induced by ischemia, which can partially reverse the down-regulation of Bcl-2 [81–83]. Koike et al. reported that knockout *ATG7* gene could inhibit ischemia-induced apoptosis activation and neuronal death [84]. Selectively knockout neuronal *ATG7* gene in mice can inhibit hypoxia-induced neuronal autophagy in multiple brain regions, and significantly reduce infarct volume [85]. In addition, studies have shown that in middle cerebral artery occlusion (MCAO) models, the neuroprotection induced by miRNA-30a down-regulation [86], physical exercise [87], electroacupuncture treatment [88, 89], and GSK-3b inhibitor [90] may be related to the enhancement of autophagy activity.

Nevertheless, contrasting evidence exists regarding the effects of autophagy in ischemic stroke. Administration of 3-MA 30 min before cerebral ischemia can up-regulate the expression of cleaved caspase-3 and aggravate neuronal cell death in ischemic penumbra area [91]. Nicotinamide phosphoribosyltransferase can promote neuronal survival through inducing autophagy via regulating TSC2–mTOR–S6K1 signaling pathway in a SIRT1-dependent manner during cerebral ischemia [92]. The administration of 3-MA and wortmannin (Akt inhibitor) can down-regulate the expression of beclin-1 and switch the mode of cell death from apoptosis to necrosis, suggesting that neuronal autophagy after ischemic injury seems to play an important neuroprotective role [93]. Rapamycin, a specific inhibitor of mTOR, can promote autophagy by increasing the expression of beclin-1 and LC3-II, which attenuates neuronal necrosis in a neonatal hypoxia-ischemia model [7, 93]. A few studies have also confirmed the neuroprotective effects of rapamycin [94, 95]. In OGD and MCAO model, 3-MA administration or *ATG7* silencing can significantly increase the release of cytochrome *c* and promote neuronal apoptosis [6]. Interestingly, studies have shown that there are gender differences in ischemia-induced autophagy [96]. In female mice brain tissue, there is a high basal level of autophagy and caspase activity after hypoxic-ischemic injury [96]. Furthermore, inhibition of autophagy can play different roles in a gender-dependent manner after ischemia. Inhibiting autophagy can reduce cerebral infarction volume in male mice and ovariectomized female mice, while increase cerebral infarction volume in female mice and ovariectomized female mice treated with estrogen replacement therapy [97].

#### Astrocytes

Astrocytes play vital roles in maintaining normal structures and functions of neurovascular units, including glutamate clearance, ion and pH homeostasis, synapse remodeling, neuroendocrine signaling, immunity/inflammation, neurotrophic factor production, vasoconstriction/vasodilation, blood brain barrier (BBB) function and neurogenesis [82, 98, 99]. Research has confirmed increased autophagosomes and autolysosomes and up-expression of beclin-1, LC3-II, cathepsin B and LAMP2 in focal cerebral ischemia model and astrocyte hypoxia model, which provided evidence for enhanced astrocyte autophagy [100, 101].

Mounting evidence has demonstrated the bidirectional functions of astrocyte autophagy after ischemia [102]. In OGD-treated astrocytes, autophagy exerts neuroprotective effects in an AMPK-dependent manner, while AMPK inhibition can significantly reduce the expression of autophagy-related proteins and eliminate the neuroprotective effects [103]. Up-regulation of p62/SQSTM1and LC3-II can be seen in astrocytes exposed to hypoxia, while 3-MA administration aggravates astrocytes injury and even death [104]. Impaired autophagosomes maturation, autophagic flux and autolysosome function can lead to astrocytes cell death under hypoxia condition [105, 106]. In rat astrocytes, rapamycin can reduce neurotoxicity of MeHg by activating autophagy [107]. Nevertheless, there are also studies confirming that astrocyte autophagy can aggravate ischemia damage [100].

#### Microglia

Microglia are important resident immune cells in central nervous system (CNS), microglia activation is an important pathological mechanism in CNS inflammatory response [108–110]. In-depth study of microglia autophagy will provide a reasonable basis for inflammatory intervention of cerebral ischemia.

The findings show that microglial autophagy plays an important pro-inflammatory role in cytokines production and neuroinflammatory response after ischemic stroke [111, 112]. Hypoxia/ischemia could lead to excessive activation of autophagy in microglia, which exacerbated neuroinflammatory damage [75]. Hypoxia could induce autophagy relying on up-regulated HIF-1 expression in microglia, and eventually lead to death of microglia [113]. 3-MA administration could inhibit inflammatory response in microglia, reduce brain edema and improve neurological deficits in focal cerebral ischemia model [113, 114]. Catechin could prevent microglia apoptosis induced by hypoxia/reperfusion-evoked autophagy through up-regulating Akt and mTOR phosphorylation [115]. Xia et al. reported that autophagic flux blockade regulated microglia phenotype through activating NF-κB and inhibiting CREB [116]. Intracerebroventricular injection of protein tyrosine phosphatase 1B (PTP1B) inhibitor could alleviate deleterious microglial activation and neuronal injury after ischemic stroke by modulating the ER stress-autophagy axis via PERK signaling in microglia [117]. Other studies have also confirmed that microglia autophagy can play important roles in promoting neuroinflammation after cerebral ischemia [118].

Nevertheless, there are still results showing that microglia autophagy has anti-inflammatory and anti-apoptotic effects. Activation of microglia autophagy induced by rapamycin could reduce the expression of inducible nitric oxide synthase (iNOS) and interleukin (IL)-6 stimulated by lipopolysaccharide [119]. Microglial PGC-1α could promote microglia autophagy and suppress neuroinflammation by reducing NLRP3 activation after ischemic stroke [120]. In photothrombotic model, significantly up-regulation of PARP14 (poly (ADP-ribose) polymerase family, member 14) in peri-infarct regions suppressed microglia autophagy via inhibiting the transcription of lysophosphatidic acid receptor 5 gene [121].

#### Oligodendrocytes

Oligodendrocytes maintain the integrity of neuronal axons and neuron functions, and are main targets of white matter damage in various CNS diseases [122]. Oligodendrocyte autophagy was closely related to demyelination in neurodegenerative diseases and spinal cord injury [123]. In Long-Evans Shaker rats with mutations in myelin basic protein (MBP), autophagy proteins expression were increased, resulting in loss of oligodendrocyte and severe demyelination [124]. Growing evidence highlights that white matter damage such as loss of oligodendrocytes and demyelination are important pathological mechanism after cerebral ischemia [125]. However, there are few studies concerning oligodendrocyte autophagy after cerebral ischemia, and further exploration is needed.

#### BMVECs

Destructed integrity and increased permeability of blood–brain barrier (BBB) are common pathological features of cerebrovascular diseases, leading to cerebral edema [126]. Brain microvascular endothelial cells (BMVECs) are connected by tight junctions to form a highly selective barrier and play vital roles in maintaining the structure and function of BBB [127]. Several research results indicate that BMVECs autophagy plays a neuroprotective role in ischemic stroke. Administration of rapamycin and lithium carbonate enhanced autophagy and significantly reduced BMVECs apoptosis, while 3-MA treatment had the opposite effect, which verified the neuroprotective effect of BMVECs autophagy on BBB integrity during I/R injury [126]. Inhibiting autophagy with chloroquine enhanced the permeability of BBB and aggravated brain edema after cerebral ischemia in diabetic rats [128]. Rapamycin could induce autophagy in BMVECs and promote cell survival after OGD insults [129]. However, other research results showed that enhanced autophagy activity contributed to BMVECs injury following hypoxic insults [130]. It was confirmed that selenium could attenuate ischemia/reperfusion injury‑induced BBB injury by PI3K/AKT/mTOR pathway‑mediated autophagy inhibition under hyperglycemia conditions [131]. In-depth exploration of BMVECs autophagy may provide potential therapy strategies for BBB neuroprotection after ischemic stroke.

Based on the current research results, it can be inferred that the complexity and heterogeneity of experimental animal species/age/sex, cell/animal models, different brain regions/cell types, intensity and duration of hypoxia/ischemia may cause differences in results. Autophagy is a double-edged sword in terms of cellular adaptive system, and its dual roles may show flexible adaptive ability. How to pursue advantages and avoid disadvantages to induce effective neuroprotection is the new research direction.

### Temporal dynamics of autophagy in ischemic stroke

Activation of autophagy by rapamycin in ischemic preconditioning and before ischemia protects neurons from death [132, 133]. However, increased autophagy after ischemia exerts different effects [45]. In an in vitro ischemia model, inhibiting autophagy 24 h prior to reperfusion markedly increases neuronal death, while autophagy inhibition significantly protects neurons from death 48–72 h after reperfusion [134]. Some reports showed that activation of autophagy protects neurons from death [94], whereas others indicate that it increases neuronal death or abolishes the neuroprotective effects of ischemic postconditioning [135]. These observations indicate that the extent and time window of autophagy would affect the efficacy of autophagy intervention in ischemic stroke. Thus, a comprehensive comparison of autophagy at different time points is necessary before being used as a therapeutic target for post-ischemic neuroprotection.

Numerous studies have shown that autophagy is activated in the penumbra following ischemic stroke [136]. Autophagy was increased in the penumbra 1 h after MCAO, of LC3-II levels steadily increased 2–5 h following ischemic stroke, reaching a maximum at 5 h [137]. During this stage, infarct volume and the severity of the neurological deficit increased slowly, suggesting that activation of autophagy might have a neuroprotective function. At 12–72 h post MCAO, LC3-II levels in penumbra rapidly decreased, suggesting that neuronal autophagy was reduced 12–72 h after ischemic stroke [137]. Interestingly, the LC3-II/cleaved caspase-3 ratio in penumbra was reduced to a very low level 24 h after MCAO, showing that the shift from autophagy to apoptosis occurs 24 h following ischemic stroke [137]. In addition, administration of 3-MA can up-regulate cleaved caspase-3 expression and promote apoptosis of astrocytes 1–4 h post OGD; however, astrocytes autophagy is significantly down-regulated within 8–24 h after OGD, suggesting that astrocytes autophagy also exerts neuroprotective effects or detrimental effects in a time-dependent manner [138].

### Crosstalk between autophagy and other pathological processes

After ischemic stroke, autophagy and a variety of other cell biological processes interact with each other to form a complex pathophysiological mechanism network, which together regulate the death or survival of nerve cells.

#### ROS

ROS is closely related to neuroinflammation after cerebral ischemia, including oxygen anions, hydrogen peroxide and free radicals [139–141]. Excessive ROS accumulation can induce autophagy mainly by regulating transcription of autophagy-related genes after cerebral ischemia [142]. ROS can increase the expression of p53 and HIF-1, which activate promoters of autophagy-related genes [143, 144]. ROS can promote the expression of p62/SQSTM1 via transcription factor NF-E2-related factor 2 (NRF2) binding to a specific motif located in the p62/SQSTM1 promoter [145]. In addition, accumulated ROS can induce the transcription of protein kinase-like ER kinase (PERK), thereby promoting the transcription of autophagy-related genes [146]. It was confirmed that autophagosomes formation increased in neonatal hypoxia ischemic brain, whereas pharmacologic inhibition of NADPH oxidase counteracted autophagy-induced neuroprotection [147].

Ischemic-induced autophagy could modulate ROS generation to resist oxidative stress damage [148]. Inhibiting autophagy with 3-MA can aggravate OGD-induced ROS accumulation and promote apoptosis of brain endothelial cells [126]. In addition, Sirtuin3 can reduce neuronal oxidative stress damage by inducing autophagy, exerting a neuroprotective effect [149]. Overall, these results prove that ischemia-induced ROS accumulation can activate autophagy, which can in turn exert anti-oxidative response to remove excessively accumulated ROS.

#### Mitochondrial dysfunction

After cerebral ischemic stroke, mitochondrial dysfunction can significantly impede ATP synthesis and lead to increased concentration of intracellular Ca^2+^ [150], which further aggravates mitochondrial damage and initiates mitophagy. Remote ischemic post-conditioning can up-regulate the expression of Parkin and promote mitophagy, thereby significantly inhibiting oxidative stress in rats MCAO models [151]. Administration of rapamycin can improve mitochondrial dysfunction and reduce cerebral infarction volume in rats MCAO models [95, 152]. There are other studies confirming the neuroprotective effect of mitophagy in ischemic stroke [153, 154].

#### ER stress

Ischemia-induced ER stress is triggered by increased Ca^2+^ concentration in ER and accumulation of misfolded proteins, which is closely related to autophagy activation [155, 156]. 3-MA can aggravate ER stress and promote the expression of pro-apoptotic proteins caspase-3 and caspase-12, which attenuates the neuroprotective effect of cerebral ischemic preconditioning [157]. Salubrinal, the ER stress inhibitor, inhibits autophagy activity and neuroprotection induced by brain ischemic preconditioning [158]. Melatonin treatment before ischemia inhibits ER stress-induced autophagy and attenuates acute neuronal injury after ischemic stroke via inositol-requiring enzyme 1α (IRE1α) and PERK signaling pathway [159]. However, other studies report that neither enhancement of autophagy nor suppression of ER stress exerts neuroprotection in neurons under ischemic stroke condition [160].

#### Neuroinflammation

Autophagy plays important roles in modulating the excessive inflammatory response [161]. It has been confirmed that ischemia can induce mitochondrial depolarization and release of ROS and mitochondrial DNA, which resulted in the formation of inflammasomes [162]. Autophagy can directly isolate inflammasomes through the autophagy-lysosome pathway or clear depolarized mitochondria through mitophagy, thereby inhibiting the synthesis of inflammasomes and reducing inflammation damage [163].

## G-protein-coupled receptors

### Classification, function and composition

GPCRs are one of the largest protein receptor superfamilies in mammals [164, 165]. Known as seven-fold helix transmembrane protein receptors, GPCRs share the commonality of an N-terminal, seven-fold transmembrane α-helix, C-terminal, three extracellular rings, and three intracellular rings [165–167]. Approximately 1200 genes in the human genome encode GPCRs, and more than 800 GPCRs have been identified based on the results of sequencing analysis [168]. Human GPCRs are divided into five major families, including adhesive frizzled/taste 2, rhodopsin (class A), secretin, glutamate and orphan receptors [167–169]. About 15% of GPCRs have not yet found endogenous ligands, which are designated as orphan receptors [169].

GPCRs recognize various extracellular signals, including neurotransmitters, peptides, hormones, biotin, glycoproteins, lipids, nucleotides, ions, proteases, light, odorants, pheromones, eicosanoids and organic compounds [165, 167, 170], which subsequently activate intracellular heterotrimeric guanosine monophosphate binding proteins. Activated G-proteins regulate second messengers to transmit extracellular signals to intracellular effector molecules [170].

Membrane receptor-coupled G-proteins mainly include heterotrimeric G-proteins and small G-proteins. The G-proteins discussed in this review refer to heterotrimeric G-proteins that are related to signal transduction. The α, β and γ subunits together compose the heterotrimeric G-proteins. β and γ subunits are closely bound in a non-covalent Gβγ dimer. Gα subunits are inactive when binding to Gβγ dimers. Gα subunits contain guanine nucleotides binding regions, which have GTPase activity. Reversible binding with GTP and GDP affords G-proteins the ability to act as molecular switches in signal transduction cascade.

### Transmembrane transduction mechanisms

The general transmembrane transduction process is divided into the following steps. Under physiological conditions, G-proteins exist as heterotrimers, Gα subunit binds strongly to GDP, and Gβγ dimer binds loosely to Gα subunit and GDP. Upon exposure to extracellular signals, GPCRs are activated and induce structural changes in Gα subunits, which possess GTPase activity and lead to the exchange of GDP to GTP. The binding of GTP with Gα subunit could result in separation of Gα subunit and Gβγ dimer. Gα subunits are activated and act on downstream effectors to generate intracellular signals.

Until now, four different Gα proteins have been identified, including Gα_q/11_, Gα_12/13_, Gα_s_ and Gα_i/o_. Among them, Gα_s_ and Gα_i/o_ coordinately regulate the biological activity of adenylate cyclase (AC). Gα_s_ can activate AC activity, while Gαi/o inhibits AC activity. AC can promote the production of 3′5′-cyclic adenosine monophosphate (cAMP), which mediates cAMP-dependent protein kinase to phosphorylate serine or threonine residues of many intracellular proteins. Gα_q/11_ activates phospholipase C (PLC), which converts phosphatidylinositol 4 and 5-bisphosphate (PIP2) to diacylglycerol (DAG) and inositol 1,4,5-triphosphate (IP3). Gα_12/13_ targets guanine nucleotide exchange factor to regulate the Ras homolog gene family (Rho), which is involved in cytoskeleton regulation.

After the dissociation of Gα subunit and Gβγ dimer, the receptor is internalized and turned into the closed state again. GTP can be hydrolyzed and Gα subunit recombined with Gβγ dimer in the presence of magnesium ions, which inactivates G-proteins.

## GPCRs-mediated autophagy

Increasing evidence suggest that nutritional deprivation can directly or indirectly activate GPCRs to mediate a series of downstream biological processes, including autophagy [11–13, 76, 170]. Recent studies have shown that GPCRs can also be degraded in an autophagy-dependent manner [171, 172]. The two-way regulatory mechanism between GPCRs and autophagy jointly participate in cellular biological processes and maintaining biological functions.

### Activation of GPCRs-mediated autophagy

#### Direct activation of GPCRs by nutritional deprivation

Certain types of GPCRs, mainly including amino acid-sensing GPCRs and fatty acid-sensing GPCRs, can be activated by directly sensing the levels of nutrients [10, 173].

#### Amino acid-sensing GPCRs

Such amino acid-responsive GPCRs mainly include Ca^2+^-sensing receptor (CaSR), GABA receptors, sweet taste receptor type 1 member 1 and 3 (T1R1/T1R3), GPRC6A, metabotropic glutamate receptors and several orphan receptors, most of which belong to the GPCR class C [173, 174]. T1R1/T1R3 have different affinities for 20 amino acids and are direct sensors of amino acid metabolism. Among them, l-glutamate level is the most sensitive signal stimulus. T1R1/T1R3 can activate mTOR protein complex 1 (mTORC1) after sensing the fluctuation of amino acid level, and regulate protein synthesis, autophagy and other biological processes [173, 175–177]. T1R3 silencing can greatly reduce the activity of mTORC1 under nutrient deprivation conditions, indicating that mTORC1 activation depends on the activation of T1R1/T1R3 by amino acid signals [177].

#### Fatty acid-sensing GPCRs

Such amino acid-responsive GPCRs mainly include long chain fatty acid receptors GPR120 and GPR40, short-chain fatty acid receptors GPR41 and GPR43, and several orphan receptors [170]. However, there is no evidence that fatty acid-sensing GPCRs are directly involved in the regulation of autophagy, but it can be speculated that they may be anticipated in the process of autophagy in view of their function in detecting nutrients under nutrient deprivation conditions [178].

### Indirect activation of GPCRs by nutritional deprivation

The fluctuation of nutrients can also indirectly activate GPCRs by promoting the secretion of neurotransmitters and hormones, and then regulate autophagy and other biological processes, mainly including β-adrenergic GPCRs and muscarinic GPCRs [170].

#### β-adrenergic GPCRs

When blood glucose level decreases, hypothalamic neurons sense the fluctuations in glucose levels and act on the adrenal glands to promote the secretion of adrenaline. Adrenaline can activate β-adrenergic GPCRs, which enhance autophagic flux by promoting the fusion of autophagosomes and lysosomes to induce lipolysis of triglycerides [179]. Inhibition of β1 adrenergic signaling via anti-β1 adrenergic receptor autoantibodies can inhibit autophagy and cause cardiac damage in rats, which can be reversed by mTOR inhibitor rapamycin [180]. In addition, the β2-adrenergic specific agonist salbutamol can increase the autophagy flux in cardiac fibroblasts [181].

#### Muscarinic GPCRs

Amino acid deprivation can increase the activation of muscarinic acetylcholine Gα_q_-coupled GPCRs, which promotes MAPK signal transduction in pharyngeal muscles and leads to increased food intake [182]. Amino acid deprivation can cause muscarinic activation, and worms lacking G-protein β subunit would die due to excessive autophagy in pharyngeal muscles [183]. In addition, hypoxia and reoxygenation treatment can induce autophagy in H9C2 cells, and acetylcholine treatment can activate muscarinic receptors to promote autophagy and ultimately increase the survival of H9C2 cells [184].

#### Other GPCRs

GLP-1 analogs can enhance autophagic flux and inhibit hepatic steatosis [185]. Intraventricular injection of liraglutide, a GLP-1R agonist, can increase the level of lipid-modified LC3-II and improve heart function in mice, suggesting that the activation of GLP-1R can promote autophagy to exert cardioprotective effects [186]. Hyperglycemia promotes the clearance of damaged mitochondria in pancreatic β cells by inducing autophagy, thereby attenuating oxidative stress injury [187, 188]. Hyperglycemia can also activate P2Y purinergic receptors through increased production of ADP [189]. In HepG2 cells, ADP can induce autophagy by activating P2Y13 receptor [190].

### GPCRs-mediated autophagy signaling pathway

**Fig. 2:**
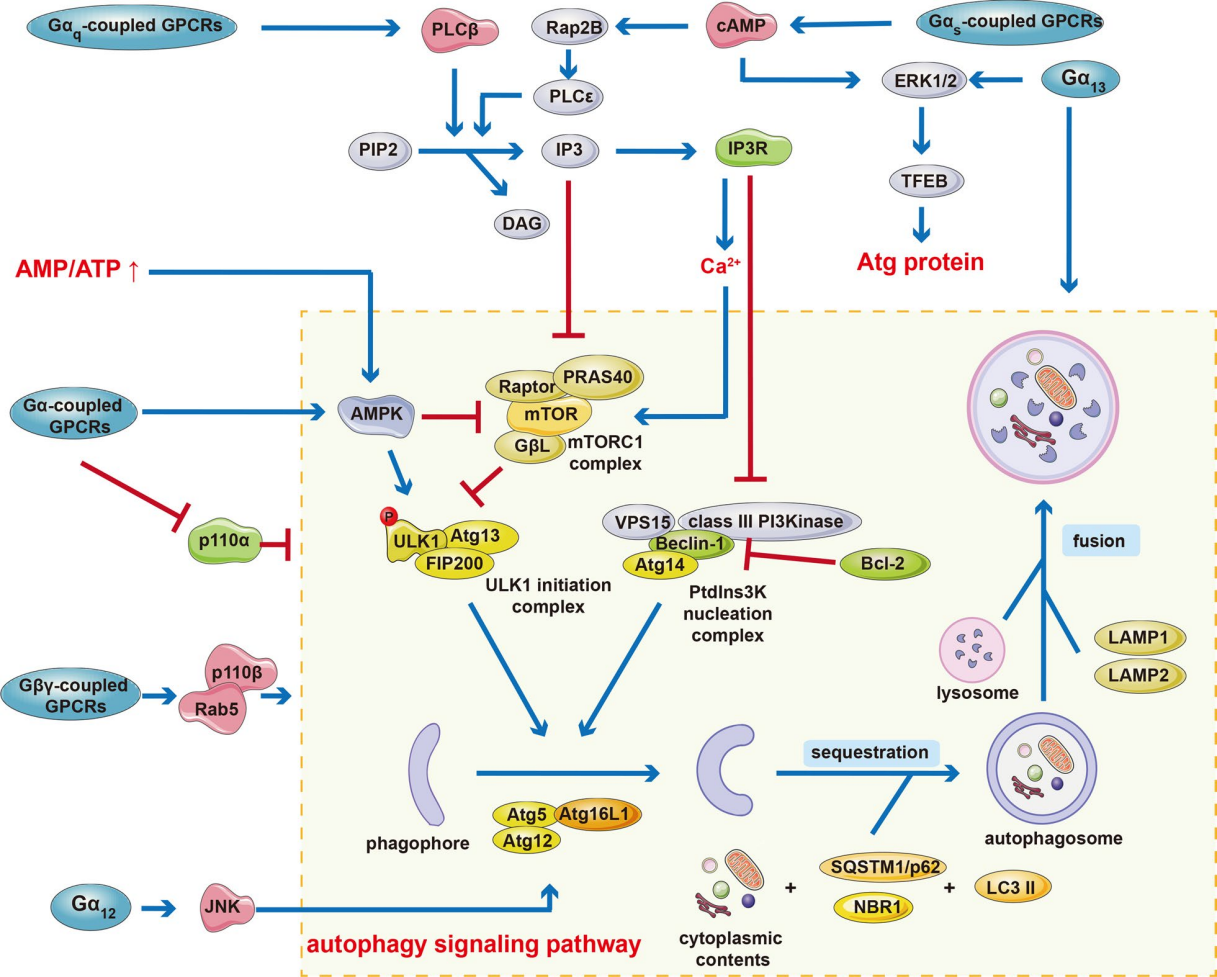
Core mechanisms of GPCRs-mediated autophagy signaling pathway. The blue oval frames represent different GPCRs subfamilies, and the core machinery of autophagy is in the yellow dashed frame. The blue arrows represent promotion, and the red arrows represent inhibition. It can be seen from the diagram that different GPCRs subfamilies regulate autophagy through multiple pathways, forming a complex regulatory network

Nutrient receptors are activated and modulate different Gα subunits to initiate various downstream signaling mechanisms to mediate autophagy (Fig. 2).

#### mTOR

The activation of mTORC1 is one of the important mechanisms of autophagy induction. More and more research results indicate that Gα_q_-coupled GPCRs (such as endothelin ET-B receptor and muscarinic M3 receptor) and Gα_s_-coupled GPCRs can activate mTORC1; while, Gα_i_-coupled GPCRs have not yet been observed to be involved in the activation of mTORC1 [177, 191, 192]. Gα_s_-coupled GPCRs increase cAMP to activate protein kinase A (PKA) and PKA phosphorylates the mTORC1 component Raptor on Ser 791, leading to decreased mTORC1 activity [11]. Bennett and colleagues reported that the activation of mTORC1 can be inhibited by the Gα_i_ subunit inhibitor pertussis toxin, suggesting that Gα_i_-coupled GPCRs can also activate mTORC1 [193]. Recent studies have shown that increased Gβγ subunits can interact with the carboxyl terminal of mTOR to mediate mTORC1 activation in yeast and HEK-293 cells [194]. It can be speculated that it is depend on the types of cells, physiological functions, and types of stress stimuli that determine which types of G-protein subunits can activate mTORC1. The activation of G protein-coupled receptor 30 (GPR30, an estrogen membrane receptor) mainly regulates the PI3K–AKT–mTOR signaling pathway to inhibit excessive autophagy induced by glutamate and protect neurons from excitotoxicity [195]. The lysosomal GPR137B can interact with Rag GTPases to regulate mTORC1 in the absence of amino acids; however, autophagy is abnormal and lysosomes are damaged when GPR137B is knocked out, indicating that GPR137B can control the location and activity of mTORC1 and maintain lysosome morphology [196].

#### cAMP

cAMP, an important second messenger, is involved in regulating many signaling pathways in cells. Studies have confirmed that the activation of Gα_s_-coupled GPCRs can increase intracellular cAMP and regulate a variety of cytological processes, including autophagy [197, 198]. In adipocyte-derived mesenchymal stem cells, prostaglandin E2 (PGE2) can activate Gα_s_-coupled EP2 and EP4 receptors to mediate the increase in cAMP production, which increases autophagic flux mainly by activating ERK1/2 [199]. In neuronal cells, cAMP can activate the guanine nucleotide exchange factor Epac, which activates Rap2B, and then Rap2B further activates PLCε, which thereby increases the level of IP3 and ultimately inhibit autophagy [200, 201]. It can be seen that there are differences in the effects of cAMP on autophagy in different cells, and further studies are needed to clarify the underlying mechanisms of the cell type-specific opposing effects.

#### PLCβ, IP3, and Ca^2+^

When amino acids are sufficient, it may activate mTORC1 in a PLCβ-dependent manner by activating the Gα_q_-coupled GPCRs to inhibit autophagy [193]. Subsequently, PLCβ cleaves PIP2 to produce IP3 and DAG, and then IP3 can regulate and inhibit autophagy in an mTORC1-independent manner [193]. Using antagonists to inhibit or gene manipulation to silence IP3R will trigger autophagy [202–204]. The result of coimmunoprecipitation between Beclin-1 and IP3R shows that after activation, IP3R can form an IP3R–Bcl-2–NAF-1 complex with anti-apoptotic protein Bcl-2 and nutritionally deficient autophagy factor 1 (NAF-1), thereby isolating beclin-1 inhibits autophagy, which indicates that the inhibitory effects of IP3R and Bcl-2 on autophagy are mechanically linked [203, 205–207]. IP3 can also activate IP3R located on the ER membrane to induce the release of Ca^2+^ from the ER. Increased intracellular Ca^2+^ can activate Ca^2+^-dependent cysteine protease and mTORC1 to reduce autophagy [200, 204]. However, other studies have shown that increased cytoplasmic Ca^2+^ induced autophagy through activating AMPK via calcium/calmodulin-dependent protein kinase β. In addition, the increased Ca^2+^ can also mediate the activation of autophagy through protein kinase C theta or ERK1/2 [208]. Further research is needed to determine the mechanism underlying opposite effects of increased cytoplasmic Ca^2+^ on autophagy in different cell types.

#### Gα_i_

Current research results have confirmed that Gα_i_ subunits, especially Gα_i3_, are involved in autophagy regulation. Gα_i3_ guanine nucleotide dissociation inhibitor and Gα_i3_ GTPase activator protein AGS3 (G protein signal transduction activator) are two important negative regulators of Gα_i3_, their activation can inhibit Gα_i3_ activity and induce autophagy [209–211]. In HT-29 cells, Gα_i3_ involved in the regulation of autophagy is essential for the transportation and metabolism of N-linked glycoproteins and sphingolipids [211–213]. This Gα_i3_-dependent autophagy regulation process mainly involves the binding of Gα_i3_ to the VPS15 regulatory subunit of the class III PI3 kinase VPS34 [214]. Studies have shown that stimulating starved hepatocytes with insulin can cause Gα_i3_ to transfer from autophagosomes to the plasma membrane and inhibit starvation-induced autophagy [215]. In Gα_i3_^−/−^ mice, the negative regulatory effect of insulin on autophagy disappears, indicating that the transformation of Gα_i3_ cytoplasmic localization may be involved in the regulation of autophagy [210]. Similarly, after phenylalanine treats hungry cells, Gα_i3_ is transferred from autophagosomes to diffuse cytoplasmic distribution, and at the same time, autophagy activity decreases [216]. These data indicate that Gα_i3_ can sensitively sense changes in hormones and nutrients and then regulate autophagy, but the exact mechanism of action has not yet been elucidated. It is speculated that Gα_i3_-GTP usually inhibits autophagy, and binding of Gα_i3_-GDP can promote autophagy.

#### Class IA PI3-kinase

Studies have shown that class IA PI3-kinase p110β is activated by directly binding to the released Gβγ subunit after GPCRs activation [217]. In the absence of growth factors, p110β can bind to Ras-related protein in brain 5 (Rab5) to mediate autophagy [218, 219]. It should be noted that the binding regions of Gβγ and Rab5 subunits on p110β are located closely, which means that their interactions may be mutually exclusive. GPCRs may also negatively regulate the p110α isoform of the class IA PI3-kinases and block its growth-promoting and anti-autophagic signals, for example, Gα_q_ and Gα_16_ subunits can inhibit p110α and attenuate autophagy activity [220–222]. This indicates that the different subunits released by the dissociation of heterotrimers after GPCRs activation may have opposite effects on PI3 kinase signaling and autophagy: the Gβγ subunit activates p110β to enhance autophagy, whereas the Gα subunit inhibits p110α to inhibit autophagy.

#### MAPKs signaling

MAPKs signaling is one of the key pathways for GPCRs to regulate autophagy, in which ERK, p38 MAPK and JNK signaling pathways all play important roles. ERK2 activation can induce Gα_i3_-dependent autophagy [223]. ERK signaling regulates the expression of autophagy-related genes by mediating the phosphorylation of growth factors and nutrient-dependent transcription factor TFEB to prevent its nuclear localization [224]. Under starvation conditions, ERK1/2 activity is reduced, and TFEB phosphorylation is enhanced to promote its nuclear localization, thereby enhancing the transcription of autophagy-related genes and lysosomal biogenesis genes, which ultimately promoting autophagy [225]. Studies have shown that ERK1/2 pathway proteins and autophagy-related proteins co-localize outside the nucleus and autophagosomes cavity, which provides direct evidence for the involvement of MAPK pathway in regulating autophagy [226]. In primary astrocytes, excessive glutamate stimulation can inhibit p38 MAPK pathway, which reduce the autophagy and promote cell proliferation [12]. When treated with the GPR30 agonist G1, autophagy can be restored to the basic level by regulating p38 MAPK pathway, suggesting that activation of GPR30 can induce autophagy via regulating p38 MAPK pathway in astrocytes [12]. The activation of pH-sensing receptor GPR68 can induce ER stress through the phospho-inositol required 1α/c-Jun N-terminal kinase (IRE1α/JNK) pathway in the intestinal epithelial cells, indicating that the pH-sensing receptor GPR68 downstream IRE1α/JNK pathway are potential treatment targets to improve inflammatory bowel disease [227]. The complex mechanism crosstalk between MAPK signaling pathway and autophagy is worthy of further exploration.

#### AMPK

Nutritional deprivation, especially an increase in AMP/ATP ratio, can activate AMPK which is an important autophagy regulatory pathway [19, 66]. On the one hand, AMPK can induce autophagy in an indirect way by reducing the activity of mTORC1. On the other hand, AMPK can directly activate specific autophagy regulatory proteins to activate autophagy [66]. Numerous receptors coupled to Gα_q/11_, Gα_s_, and Gα_i_ activate AMPK [228]. Further work is needed to determine the relationship between GPCRs-induced AMPK activation and autophagy regulation.

#### Other signaling molecules

Ligand-dependent activation of GPR119, which is mainly coupled to Gα_s_ signaling, stimulates the secretion of insulin and the expression of GLP-1 in the intestine to reduce blood glucose levels [229]. In breast cancer cells, GPR119 agonists reduce mitochondrial oxidative phosphorylation and stimulate glycolysis, leading to excessive production of lactate that can inhibit the formation of autophagosomes [230]. Frontotemporal lobar degeneration (FTLD), second in prevalence only to Alzheimer’s disease as the cause of nonvascular dementia, displays an accumulation of hyperphosphorylated tau within inclusion bodies of neurons in the cortex and temporal lobes. Monomeric β-arrestin2 binds to GPCRs and regulates GPCRs desensitization and internalization in healthy neurons; however, in FTLD, β-arrestin2 dissociates from GPCRs, and increased oligomeric β-arrestin2 impairs tau clearance and promotes tau aggregation by impeding the autophagy cargo carrier p62/SQSTM1 [231]. Hence, β-arrestin2 oligomer-to-monomer transition may function as a regulatable molecular switch to toggle p62-mediated autophagy. In mammalian cells, Atg14L/Barkor, Vps34, the catalytic subunit of the class III PI3K, Beclin 1 and p150 can combine together to form the PtdIns3K complexes which are of significant importance in the membrane isolation when the autophagosomes formation initiates [232]. Zinc finger and BTB domain containing 16 (ZBTB16), also named as Zfp145 or promyelocytic leukemia zinc finger, can mediate the binding of Cullin 3 (CUL3) to form CUL3-ZBTB16 complexes [233]. CUL3-ZBTB16 complexes can mediate the proteasomal degradation of Atg14L to regulate autophagy, which is controlled by GPCRs ligands through glycogen synthase kinase-3β phosphorylation [234]. Chemotactic GPCRs, including the C-X-C chemokine receptor CXCR4 and the urotensin 2 receptor UTS2R, can prevent the formation of pre-autophagosomal and induce a marked reduction in the autophagosomes formation via the activation of calpains in both U87 glioblastoma and HEK-293 cells, which favors the formation of adhesion complexes to the extracellular matrix and promotes chemotactic migration [235].

### Autophagy-mediated GPCRs regulation

After activation induced by specific ligands, the internalized GPCRs mainly experience two different fates, which result in different outcomes on cellular signal transduction [236]. Internalized GPCRs can be sorted and recycled, which leads to rapid signal re-sensitization. In addition, internalized GPCRs can also be transported and sequestered to the lysosomes for degradation, which leads to permanent signal transduction termination [236]. The most extensively studied mechanism involved in endocytic GPCRs being transported and segregated to lysosomes is the endosome sorting complex transport mechanism (ESCRT), which requires endocytosed multivesicular bodies to participate in the delivery of targeted ubiquitinated GPCRs to lysosomes [237]. GPCR-related sorting protein 1 (GASP1) and GASP2 are involved in the lysosomal degradation of certain GPCRs through an ESCRT-independent mechanism [238]. Such GPCRs include delta opioid receptor, dopamine D2 receptor, β-adrenergic acyclic mutant receptor, cannabinoid 1 receptor and bradykinin 1 receptor [238]. Beclin-2, similar to beclin-1, can interact with class III PI3K complexes and Bcl-2, and play a role in the lysosomal degradation pathway, thereby participating in autophagy regulation [172]. Studies have shown that the lysosomal degradation of a small number of GPCRs depends on the interaction between beclin-2 and GASP1 [171]. Mutations at key loci of beclin-2 can block its binding with GASP1, which results in hindrance of the lysosomal degradation of GPCRs, suggesting that the interaction between beclin-2 and GASP1 is necessary for GPCRs degradation [171]. Upon food deprivation, hepatic G protein-coupled receptor kinase 2 (GRK2, a Ser/Thr kinase that classically known for its role in regulation of GPCRs via β-arrestin-dependent desensitization and the uncoupling of G proteins) levels decrease abruptly in an autophagy-dependent manner in primary hepatocytes and in mice models, promoting glycogenolysis and gluconeogenesis and thus enhancing glucagon-mediated metabolic cascades [239].

## GPCRs-mediated autophagy in ischemic stroke

At present, researchers generally believe that autophagy provides adaptive neuroprotection against cerebral ischemia and maintain homeostasis of the brain tissue after ischemic stroke. GPCRs are the most fruitful type of receptors in drug research, and their importance has been widely recognized in the academic and pharmaceutical fields. As mentioned above, GPCRs-mediated autophagy and the mechanisms of autophagy activation after ischemic stroke overlap substantially. The roles and mechanisms of GPCRs-mediated autophagy after ischemic stroke remain unclear. Therefore, research on GPCRs-mediated autophagy and the clinical relevance of their interplay in the pathogenesis of ischemic stroke are of considerable significance.

GPR37 is mainly expressed in multiple brain regions including striatum and hippocampus, especially in mature oligodendrocytes [13, 240, 241]. After ischemic stroke, the down-regulation of GPR37 can induce autophagy via inhibiting the mTOR signaling pathway [13]. In addition, the down-regulation of GPR37 can promote microglia activation and transformation to M1 phenotype, and disrupt the glial scars formation in cortex around the infarct core, thereby aggravating cell damage [13]. The above research results indicate that GPR37 is a potential target for regulating inflammatory response and neuroplasticity after ischemic stroke.

GPCRs are negatively regulated by a class of GTPases termed regulator of G-protein signaling (RGS) [242]. RGS5, a potent negative regulator of Gα_q_ and Gα_i_, structurally consists of a conserved RGS domain that binds to the corresponding Gα subunit and dephosphorylates the active GTP-bound Gα subunit via the GTPase-stimulating activity of the RGS domain [243, 244]. RGS5 stabilizes and maintains BBB integrity after focal cerebral ischemia via the inhibition of Gα_q_ and its coupled receptors, mGluRs and NMDARs, resulting in attenuation of ROCK and MLCK signaling pathways, which affect actin cytoskeletal reorganization, endothelial tight junctions, cell permeability, and stroke severity [244, 245]. Indeed, RGS5 maintains brain endothelial cell function in focal cerebral ischemia. Several studies on GPCRs-mediated autophagy signaling pathway in ischemic stroke have been reported, and this pathway has demonstrated positive prospects as a novel mechanism for treating ischemic stroke. A comprehensive understanding of GPCRs-mediated autophagy signaling pathway in ischemic stroke will provide novel directions in the search for therapeutic drugs for ischemic stroke.

## Conclusions and clinical perspectives in neuropharmacology

At present, the dual roles and underlying mechanisms of autophagy in ischemic stroke have attracted more and more attention. After ischemic stroke, autophagy plays dual roles in a time-dependent and cell-specific manner, therefore, comprehensive consideration of activating or inhibiting autophagy to achieve neuroprotection, while avoiding the harmful effects is the direction of future research. In addition, due to the extensive roles of autophagy in many cellular functions, inhibition or inappropriate activation of autophagy may not be ideal. There are interactions between the mechanism of autophagy and the signal pathway of GPCRs, which provides research evidence for regulating GPCRs-mediated autophagy to exert neuroprotection after ischemia. Further studies on the GPCRs-mediated autophagy signaling pathways after ischemic stroke are warranted. GPCRs are excellent drug targets, accounting for nearly one-fifth of all commercially marketed drugs that act by binding to GPCRs. The development of new drugs or the use of existing drugs to target specific GPCRs that regulate autophagy after ischemic stroke may have significant therapeutically potential in the treatment of ischemic stroke.

## Data Availability

Not applicable.

## Abbreviations

GPCRs: G-protein-coupled receptors
CMA: Chaperone-mediated autophagy
HSPA8/HSC70: Heat shock 70 kDa protein 8
LAMP2A: Lysosome-associated membrane protein 2A
Atg: Autophagy-related proteins
PAS: Pre-autophagosomal structures
AMP: Adenosine 5ʹ-monophosphate
mTOR: Mammalian target of rapamycin
ULK1: Unc-51-like kinase 1
AMPK: Adenosine monophosphate-dependent protein kinase
LC3: Microtubule-associated protein light chain 3
HIF-1: Hypoxia inducible factor 1
BNIP3: Bcl-2/adenovirus e1b-interacting protein 3
α7nAChR: α7 Nicotinic acetylcholine receptor
NF-κB: Nuclear factor-kappa B
IKK: IκappaB kinase
PI3K: Phosphoinositide 3-kinase
3-MA: 3-Methyladenine
BMVECs: Brain microvascular endothelial cells
OGD: Oxygen glucose deprivation
siRNA: Small interfering RNA
BFA: Bafilomycin A1
MCAO: Middle cerebral artery occlusion
PDE1-B: Phosphodiesterase enzyme 1-B
PTP1B: Protein tyrosine phosphatase 1B
PARP14: Poly (ADP-ribose) polymerase family, member 14
iNOS: Inducible nitric oxide synthase
IL: Interleukin
PGC-1α: Peroxisome proliferator-activated receptor γ coactivator-1α
NLRP3: NOD-like receptor thermal protein domain associated protein 3
a-syn: A-synuclein
BBB: Blood–brain barrier
BMVECs: Brain microvascular endothelial cells
ER: Endoplasmic reticulum
NRF2: NF-E2-related factor 2
PERK: Protein kinase-like ER kinase
IRE1α: Inositol-requiring enzyme 1α
Camp: Cyclic adenosine monophosphate
PLC: Phospholipase C
PIP2: Phosphatidylinositol 4,5-bisphosphate
DAG: Diacylglycerol
IP3: Inositol 1,4,5-triphosphate
Rho: Ras homolog gene family
GRKs: GPCR kinases
CaSR: Ca2^+^-sensing receptor
T1R1/T1R3: Sweet taste receptor type 1 member 1 and 3
mTORC1: MTOR protein complex 1
MGL: Metabotropic glutamate receptors
PUFA: ω-6 Polyunsaturated fatty acids
GLP-1: Glucagon-like peptide 1
GPB-2: G protein b subunit
GLP-1R: Gs-coupled receptor
PKA: Protein kinase A
PGE2: Prostaglandin E2
NAF-1: Nutritionally deficient autophagy factor 1
AGS3: GTPase activator protein
Rab5: Ras-related protein in brain 5
MAPK: Mitogen-activated protein kinase
IRE1α/JNK: Phospho-inositol required 1α/c-Jun N-terminal kinase
FTLD: Frontotemporal lobar degeneration
ZBTB16: Zinc finger and BTB domain containing 16
CUL3: Cullin 3
ESCRT: Endosome sorting complex transport mechanism
GASP: GPCR-related sorting protein
GRK2: G protein-coupled receptor kinase 2
RGS: Regulator of G-protein signaling
